# Towards Improved Measurement of Financial Protection in Health

**DOI:** 10.1371/journal.pmed.1001087

**Published:** 2011-09-06

**Authors:** Rodrigo Moreno-Serra, Christopher Millett, Peter C. Smith

**Affiliations:** 1 Business School and Centre for Health Policy, Imperial College London, London, United Kingdom; 2 Department of Primary Care and Public Health, Imperial College London, London, United Kingdom; 3 South Asia Network for Chronic Disease, Public Health Foundation of India, New Delhi, India

## Abstract

Christopher Millett and colleagues argue that new metrics are needed to better inform policy development on financial protection in health.

**Summary Points**

- Most health systems fail to offer adequate financial protection to citizens because of insufficient financial risk pooling and prepayment mechanisms.
- The harm caused by inadequate financial protection goes well beyond that measured by conventional indicators such as catastrophic and impoverishing health spending.
- A broader set of metrics is required to better inform policy development on financial protection, including new indicators that identify citizens who cannot afford to use health services and may have very low or no health spending.
- Options include expanding the use of household surveys that assess cost barriers to health care access and the calculation of “need-adjusted” estimates of medical care utilization and spending.

Protecting citizens against the financial consequences of illness has long been a key objective of health systems worldwide. In the United Kingdom for example, financial protection—which refers to how far people are protected from the financial consequences of illness—was the fundamental goal when the National Health Service was established in 1948, more than health improvement or equitable access to health care [1]. The World Health Report 2000 identified financial protection against the costs of ill health as a fundamental objective of health systems, on the basis of the premise that a fair health system ensures households make health care payments according to their ability to pay rather than risk of illness [2]. This report helped put financial protection at the forefront of health policy and academic debates, leading to numerous studies concerned with identifying the determinants of financial protection levels [3].

Ten years later, the World Health Report 2010 called on all countries to take concrete steps towards achieving universal health coverage, defined as providing all people with access to needed health services of sufficient quality to be effective, without financial hardship associated with their use [4],[5]. This call was made because most health systems still fail to offer adequate financial protection because of insufficient financial risk pooling and prepayment mechanisms. Financial risk pooling involves the collection and management of health revenues from all members of the pool, such that the risk related to health care payment is borne collectively rather than from each individual contributor. Health systems with higher prepaid funds for health care—that is, funds paid by individuals before the event of illness, through social health insurance contributions or taxes—are likely to enhance financial protection by favoring effective spreading of financial risk across all population groups (see Box 1 for a definition of key concepts). Financial hardship due to medical payments has been estimated to affect 150 million people globally each year, in both richer and poorer countries [6]. Affected individuals face a great risk of being driven into poverty because of health care expenses, resulting also in lack of access to needed care owing to inability to pay. Nevertheless, the squeeze on public finances associated with the global economic downturn has led some governments (e.g., Spain and Greece) to increase direct payments for health services. This increase is taking place despite accumulating evidence about the detrimental effect on both financial protection and the welfare of citizens of heavy reliance on user payments for financing health systems (leading to growing calls for the complete abolition of user fees in health care) [3],[6]–[8]. In this context, service coverage and financial risk protection have been a primary topic of discussion at the WHO 64th World Health Assembly, whose resolution recommends that the matter be further debated at the forthcoming session of the United Nations General Assembly [9].

**Box 1. Glossary**

**Financial Protection**

Financial protection refers to how far people are protected from the financial consequences of illness. A growing body of research has focused on the extent to which health payments are **catastrophic** or **impoverishing**. These financial protection indicators provide information on the number of households spending a large proportion of their income on medical bills, where “large” means that their health payments exceed some threshold measured in terms of household income after subsistence costs such as food and shelter have been met (catastrophic spending), or push households below a predefined poverty line (impoverishing spending). The choice of threshold above which health care payments are defined as catastrophic or impoverishing is unavoidably arbitrary and ultimately a normative choice, varying across studies [3],[6],[16].

**Health System Financing**

The financing of most health systems include some **pooling** arrangement, meaning that financial resources are accumulated and managed so as to share the financial risks of illness across all members of the pool. Thus, members of the pool who need to use health services will not have to bear the corresponding costs all by themselves, as costs will be shared across all pool members, making ill individuals less likely to be deterred from seeking care because of health payments and less likely to fall into poverty should any payments need to be made. Higher proportions of **prepaid funds** for health care—that is, funds paid by individuals before the event of illness or injury, through channels such as social health insurance contributions or taxes—are likely to enhance financial protection by favoring effective spreading of financial risk across all population groups [4]. On the other hand, financial protection will tend to be poorer if **out-of-pocket payments** (also referred to as **cost-sharing** or **user fees**) make up for a significant share of health funding: these are payments made directly by individuals to health providers at the time medical services are provided and include, for example, fees paid for consultations, laboratory tests, hospital admissions, and drugs.

Financial protection should remain a key objective of any health system. However, we argue that there is an urgent need to develop indicators capable of providing a broader picture of financial protection for better health policy guidance, because the harm caused by inadequate financial protection in many parts of the world goes well beyond that measured by conventional indicators. In this paper, we discuss the shortcomings of conventional metrics and make recommendations about how financial protection analyses can be undertaken in a more rounded manner to better aid policy making.

## Conventional Indicators of Financial Protection Have Important Shortcomings

Conventional indicators of financial protection assess whether people suffer financial hardship in paying for health services and are based on the notions of “catastrophic” and “impoverishing” health care spending, which relate health expenditures to some threshold in terms of living standards (Box 1). These measures have noteworthy limitations. Specifically, catastrophic and impoverishing spending metrics are constructed solely on the basis of out-of-pocket medical expenditures reported in surveys, thus ignoring the fact that poorer individuals often cannot afford to use health services and therefore report very low or no health spending. As a result, these individuals will often be included amongst those considered to be protected against financial catastrophe. Whilst the presence of cost barriers to access may be broadly linked to concerns about equity of access, it is also a crucial indicator of financial protection in a health system. In most societies, a person who is unable to seek necessary care because its costs exceed their capacity to pay would not be considered financially protected. It has been estimated that most of the 1.3 billion poor citizens around the world have restricted access to health services because of cost [4],[10]. And the problem is not confined to low-income countries without effective public insurance schemes. Recent survey data from high-income countries have shown that around one-third of US adults did not get recommended care, did not see a doctor when sick, or failed to fill/skipped prescriptions because of costs, with very high proportions also observed in countries with de jure universal coverage such as Germany (25%), Australia (22%), Canada (15%), New Zealand (14%), and France (13%) [11].

The World Health Report 2010 recommended that financial protection indicators based on household out-of-pocket spending should be complemented with information on de facto coverage levels for some “key interventions,” so as to provide “clues on the extent to which financial barriers prevent people from using services” [4] (p.10). This sensible suggestion highlights the fact that current catastrophic and impoverishing spending metrics are unable to offer a complete picture of risk protection levels. Worse still, we argue that misleading policy conclusions can be obtained by focusing solely on these conventional measures.

**Figure 1:**
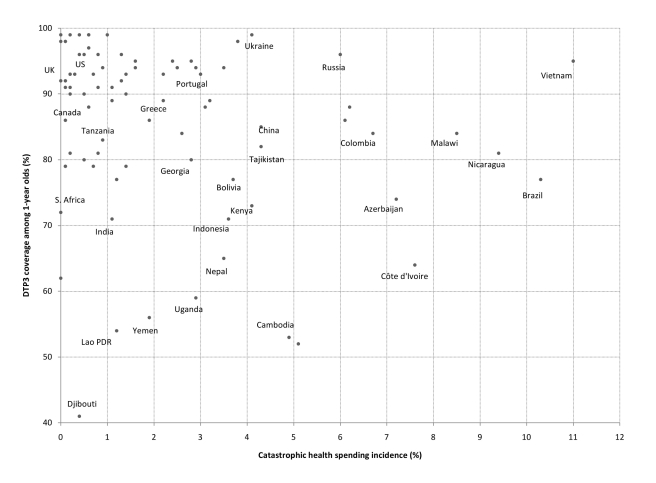
Catastrophic spending incidence and DTP3 immunization coverage among 1-year-olds, 87 countries (various years). Catastrophic spending is defined as out-of-pocket payments for health reaching at least 40% of a household's nonsubsistence income [6],[26].

**Figure 2:**
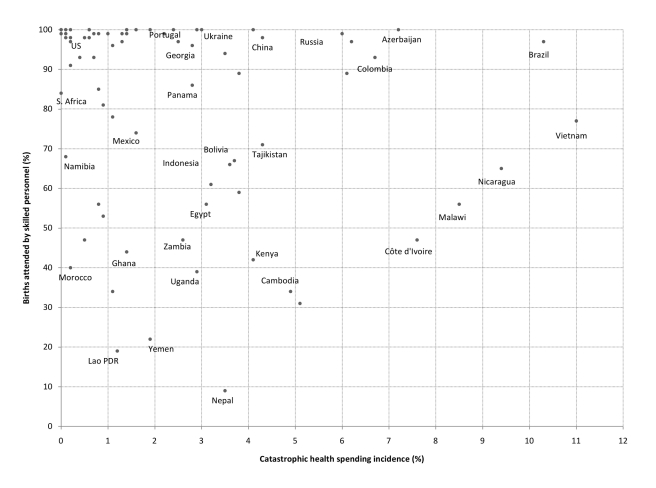
Catastrophic spending incidence and percentage of births attended by skilled personnel, 79 countries (various years). Catastrophic spending is defined as out-of-pocket payments for health reaching at least 40% of a household's non-subsistence income [6],[26].

Consider a simple illustration examining two health coverage indicators widely used by international agencies: diphtheria-tetanus-pertussis (DTP3) immunization and births attended by skilled personnel. Figures 1 and 2 present comparisons of catastrophic spending incidence and the corresponding national coverage levels for these interventions. The figures show that, for a given level of financial catastrophe incidence, there are remarkable discrepancies in coverage levels, suggesting important differences also in the presence of financial and other barriers to access across health systems at least as far as primary care is concerned. For example, the estimated incidence of catastrophic spending among households in Uganda was 2.9% in 2003, a similar figure to that estimated for countries such as Greece (2.2%) and Portugal (3.0%) [6]. A sole focus on reported catastrophic expenditures could lead an observer to conclude that citizens of Uganda enjoyed a similar level of financial protection as their counterparts in Greece and Portugal, despite the much improved breadth (universality of health benefits) and depth (lower cost-sharing) of coverage by public health insurance schemes observed in the two OECD countries [12]. In fact, empirical evidence on barriers to health care use in Uganda strongly suggests that a large share of its population is not adequately protected against the financial consequences of illnesses (in many areas such as obstetric and postnatal services, child health, and curative care), having to forgo necessary medical treatment because of costs [13]. From Figure 1 we can see that only about 59% of 1-year-olds were immunized against DTP3 in Uganda, compared to around 90% in Portugal and Greece.

Such discrepancies in coverage and access to care between countries with similar catastrophic spending levels are common even when comparisons are made within groups of similar national income per capita [14]. For instance, lower middle-income countries such as China, Ukraine, Bolivia, and Indonesia have estimated financial catastrophe incidence of about 4%. However, coverage figures for DTP3 immunization and the share of births attended by skilled personnel tend to be much higher in the former two countries (Figures 1 and 2). Analogously, Tajikistan, Kenya, and Nepal, three low-income countries with catastrophic health spending incidence between 3.5% and 4.3%, also exhibit remarkable differences concerning coverage of births attended by skilled personnel (71%, 42%, and 9%, respectively) and DTP3 immunization (82%, 73%, and 65%).

Evidence from a study by Schoen and colleagues shows that differential—and substantial—impacts of financial barriers to access are also present among richer countries with very low estimated incidence of financial catastrophe (0.5% or less) [11]. It found that 33% and 25% of individuals in the US and Germany (respectively) reported having been deterred from seeking necessary health care because of costs, against 10% in Sweden and only 5% in the United Kingdom. This finding suggests that citizens living in this group of countries are not equally protected against the financial consequences of medical needs, despite negligible levels of catastrophic spending.

## Financial Protection Measures: Suggested Areas for Development

There is an emerging consensus on the need for practical alternatives to account for the effect of cost barriers to health care access in financial protection analyses [3],[4],[6],[15]. As explained above, one alternative to obtain a broader picture of financial protection levels across and within health systems would be to complement conventional measures with information provided by de facto coverage indicators. Taking this route has a number of limitations, however. First, from a purely practical viewpoint, the coverage indicators available for such analyses may vary considerably in quality across countries and often pertain to data on selected primary care interventions (e.g., immunization rates) available from international agencies. These indicators may be more pertinent in low-income settings, posing problems if the final goal is to make international performance comparisons across health systems. Second, coverage indicators have limited scope by construction, offering little information on many other potential dimensions of forgone care (e.g., adherence to secondary prevention medications) which may result in delayed care and greater costs to individuals and health care systems. Also, there is usually scant information available at the national level on utilization figures for more complex outpatient and inpatient care—often associated with important cost barriers to access and higher likelihood of financial catastrophe [16],[17]. Finally, any analyses focusing on financial barriers to health care utilization must account for the fact that coverage measures can be influenced by a number of other nonfinancial factors such as cultural issues, workforce shortages, and health system planning [18],[19].

Surveys containing questions on patterns and reasons for foregone health care utilization seem a more attractive—and frequently feasible—alternative to complement the information from conventional financial protection indicators. Standardized, multipurpose surveys have been applied in many countries and offer useful data for financial protection assessments. The Commonwealth Fund International Health Policy Surveys, conducted in various high-income countries, are a good example [20]. They provide cross-country information on the prevalence of financial barriers to access, such as whether individuals have been deterred from using health services or following the adequate course of treatment because of costs. Several middle- and low-income countries already implement regular household surveys containing questions about effective access to care [21], and including such questions into other national surveys should not result in major additional data collection costs. Moreover, a number of household surveys containing access and utilization questions have been conducted in the past under WHO's World Health Survey project and the World Bank's Living Standards Measurement Study [22],[23]. The application of these surveys on a routine basis to allow the monitoring of the extent of financial (and other) barriers to access across countries remains an important challenge for timely, evidence-based policy.

A methodologically different route would be to attempt to incorporate the effect of financial barriers to access directly into the construction of financial protection metrics. In this regard, a small number of studies have computed “need-adjusted” figures of medical care utilization and spending using survey data, indicating the amount of medical care individuals would have received had they been treated as other individuals with similar “health need” characteristics (i.e., observable factors such as age and gender) were on average treated in the population [10],[16]. These studies indicate that policy recommendations for achieving financial protection may be very different when the effect of financial barriers to access is explicitly considered (Box 2).

**Box 2. Cost Barriers to Health Care Access and Their Consequences for Financial Protection Policy: The Case of Indonesia**

A study conducted by Pradhan and Prescott estimates the distribution of “needed” individual medical expenditures to simulate the impact of different health service price subsidy regimes on catastrophic spending incidence in Indonesia, for overall expenditures and disaggregated by self-treatment, outpatient, and inpatient care [16]. Their main conclusions are that Indonesian price-subsidization policies at the time reduced households' exposure to financially catastrophic health shocks, and that regimes of free outpatient care or publicly subsidized prices (for both outpatient and inpatient care) would be the most effective alternatives to further reduce the overall incidence of catastrophic health payments. These recommendations arise as a direct result of adjusting, in the estimations, the reported spending figures for the effect of deterred access to needed care, especially by the poorest households. The resulting policy guidance could have been markedly different had need-unadjusted spending figures been used instead. Specifically, the authors report a 37% incidence of zero health expenditures in the Indonesian survey data, with the poorest quintile of income consuming on average 1.5 times fewer outpatient services than the richest quintile, and almost five times less inpatient days. Since the observed (unadjusted) incidence of financial catastrophe due to health payments is concentrated amongst the richer households, and caused mainly by their use of inpatient services, price policies of free inpatient care would likely seem more effective in reducing reported catastrophic payments overall than outpatient care subsidization. Yet this would ignore the evidence that the Indonesian poor tend to consume both less outpatient and inpatient care because of an inability to pay, and that outpatient services utilization may frequently be financially catastrophic for the poor but not for the rich.

Both alternatives discussed above have methodological challenges of their own. Multipurpose household or health surveys with detailed medical spending information have rarely been conducted in poorer countries on a routine and relatively standardized basis, making international comparisons less straightforward. On the other hand, the usefulness of “need-adjusted” health expenditures for policy-making would depend, among other factors, on an accepted (and operational) definition of what “necessary medical spending” means in any given context. Finally, financial protection metrics should ideally take other fundamental issues into consideration, such as the long-term financial consequences of strategies frequently adopted by households to cope with medical bills (e.g., borrowing money or selling assets) [17].

Although tackling the obstacles to developing better methods of financial protection assessment is far from trivial, the payoff in terms of improved health policy-making make it worth the challenge. Sound financial protection analysis is crucial for correctly identifying those individuals at greater risk of falling into poverty or being deterred from seeking necessary care because of health payments. As such, it can provide valuable information about the potentially harmful effects of user charges in health care often implemented by governments under financial strain (including the distribution of their impacts across population groups). Accurate information from such analyses may also be used to identify those health interventions that should be given priority for public funding, on the basis of their financial protection benefits in addition to conventional resource allocation criteria such as cost-effectiveness rankings [24],[25].

## Conclusions

Most health systems fail to offer adequate financial protection to citizens. The adverse consequences of inadequate financial risk protection in health are likely to be understated in most national settings, possibly to a considerable extent. This is because conventional measures of financial protection (catastrophic and impoverishing health spending) provide no information on those citizens who cannot afford to use health services and have low or no health expenditures. The use of these conventional metrics in isolation as guiding tools may result in erroneous policy decisions. There is therefore a clear and urgent need to develop better metrics of the level of financial risk protection for sound policy-making in health systems.

## Abbreviations

DTP3: diphtheria-tetanus-pertussis
